# Psychoactive substance use and its predictors among commercial tricycle operators in Jos north local government area of Plateau State

**DOI:** 10.4103/jomt.jomt_44_19

**Published:** 2020-05-20

**Authors:** Tolulope O. Afolaranmi, Z. I. Hassan, O. J. Ugwu, M. A. Onche, J. C. Obasi, O. G. Stephen, K. G. Ugwu, P. W. Bupwatda

**Affiliations:** 1 Department of Community Medicine, University of Jos and Jos University Teaching Hospital, Jos Plateau State, Nigeria; 2 Department of Community Medicine, Abubakar Tafawa Balewa University, Bauchi, Bauchi State, Nigeria; 3 Ginza Medical Centre, Jos Plateau State, Nigeria; 4 College of Health Sciences, University of Jos, Jos Plateau State, Nigeria; 5 Department of Community Medicine, Jos University Teaching Hospital, Jos Plateau State, Nigeria

**Keywords:** Pattern, prevalence, predictors, psychoactive substance, tricycle operators

## Abstract

**Background:**

According to United Nations Office on Drugs and Crime (UNODC) around 243 million people, aged 15–64 consumed an illicit substance making this a public health problem of global dimensions with attendant physical, social, and psychological problems. Studies have shown that 59.5% of road traffic accident among commercial tricycle operator has been associated with the use of psychoactive substances. Hence, this study was conducted to assess the prevalence and pattern of psychoactive substance use and its predictors among tricycle operators in Jos north local government area of Plateau State.

**Methodology:**

This was a cross sectional study conducted among 220 commercial tricycle operators selected from a cluster of five operational units of Tricycle Riders Union using quantitative data collection instrument consisted of three sections through an interviewer’s administration method.

**Results:**

The mean age of the respondents in the study was 34 ± 10 years with the prevalence of current use of psychoactive substance being 43.2%. The predictors of psychoactive substance use were tertiary level of education (AOR = 0.06; 95% CI = 0.0074–0.4806) and family history of use of psychoactive substance (AOR = 3.30; 95% CI = 1.7164–6.3611).

**Conclusion:**

This study has demonstrated a high level of illicit psychoactive substance use among commercial tricycle operators with higher level of education negatively influence its use and a positive family history potentiating it.

## INTRODUCTION

A psychoactive substance is a chemical that stimulates the central nervous system with the ability to alter brain function, resulting in temporary changes in perception, mood, consciousness and behavior.^[1]^ According to United Nations Office on Drugs and Crime (UNODC) around 243 million people, aged 15–64 consumed an illicit substance making this a public health problem of global dimensions with attendant physical, social and psychological problems.^[1,2]^ Furthermore, it has been reported that about 1 in 200 persons experienced difficulty in regulating substance use accounting for about 27 million users globally with Nigeria accounting for the highest seizures of cannabis and other illicit drugs in Africa closely followed by Egypt.^[2]^ In recent years, commercial tricycle operation (Keke) has come to bridge the huge public transport gap in most cities across Nigeria with majority of the operators not exempted from illicit substance use which could pose significant serious danger to the operators themselves, their passengers and other road users. Studies have shown that 59.5% of road traffic accident among commercial tricycle operator has been associated with the use of psychoactive substances.^[3,4,5]^ In view ofthis, it became imperative to assess the prevalence and pattern of psychoactive substance use and its predictors among tricycle operators in Jos North Local Government (LGA) Area of Plateau State, Nigeria .

## MATERIALS AND METHODS

### Study setting

The study was carried out among tricycle operators in Jos North LGA of Plateau state, which is a cosmopolitan setting divided into four districts and twenty wards.^[6]^ Jos North LGA is bounded in the North by Toro LGA in Bauchi state, in the south by Jos South LGA, in the east by Jos east LGA and in the west by BassaLGA occupying an area of 286 sq km with a population of 429 300.^[6]^ The Tricycle Riders Union of Nigeria, Plateau State chapter was inaugurated in 2010. In Jos North Local Government Area, the Tricycle Riders Union of Nigeria has about fifty units, with each unit consisting of at least 50 registered members who are mostly male young adults.

### Study population

The study population consisted of commercial tricycle operators 18 years and above working in Jos North LGA registered with the tricycle riders union of Nigeria (TRUN) Plateau State chapter.

### Sample size determination

The sample size for this study was determined using the appropriate sample size determination formula for a cross sectional study.^[7]^ Where n is the minimum sample size, Z is the standard normal deviate at 95% confidence interval (1.96), q is the complementary probability (1–p), d is the precision of the study set at 0.05 and p is the prevalence of psychoactive substance use (Galangi—local stimulant)from a previous similar study conducted in Kano metropolis Nigeria (14.8%).^[8]^ This gave a minimum sample size of 213 after addition of 10% to cater for non, poor and incomplete responses.

### Study design

This was a cross sectional study conducted to assess the level of and pattern of psychoactive substance use and its predictors among commercial tricycle operators in Jos North LGA using quantitative method of data collection.

### Criteria for inclusion in the study

All consenting commercial tricycle operators 18 years and above registered with the Tricycle Riders Union of Nigeria Plateau State chapter operating in Jos North LGA were included in the study.

### Sampling technique

From the list of the fifty operational units of Tricycle Riders Union of Nigeria Plateau State chapter operating in Jos North LGA five units were selected through computer generated table of random number using Winpepi statistical software giving rise to the selection of Dilimi, Langtang street, Bauchi road, Old Bukuru park and Tafawa Balewa units for the study. Following which each of the units selected were taken as a cluster and all the eligible commercial tricycle operators in each who consented to participate in the study were sampled.

### Data collection tool

A semi structured interviewer administered questionnaire adapted from a previous study was used.^[9]^ The data collection instrument consisted of three sections; demographic characteristics of the respondents, level and pattern of psychoactive substance use and factors influencing use of psychoactive substance. Three research assistants were trained on the content and method of administration of questionnaire prior to the commencement of the study by the principal researcher. The data collection instrument was translated to Hausa language and back translated to English Language by two different persons proficient in Language translation to enable standardization of administration in both Hausa and English Languages. The data collection instrument was pretested in one tricycle operation unit in Jos South LGA.

### Grading of response

Lifetime and current use of psychoactive substance was assessed as used if the respondents had ever used or presently using any of alcohol, cigarette/tobacco, marijuana, heroin, opioids (codeine or tramadol), kolanut coffee/caffeine and or a locally made inhalant called “solution” singly or in combination.

### Data analysis

The data obtained were processed and analyzed using IBM SPSS version 20 where socio-demographic characteristics of the respondents were expressed in frequency and percentage. Mean and standard deviation were used as summary indices for age, duration of working per day, age at substance use debut of the respondents respectively. Crude and adjusted odds ratios as well as 95% confidence interval were used as point and interval estimates of the measure of effect of the predictors on use of psychoactive substance in the logistic regression model.

Furthermore, a probability value of less than 0.05 was considered statistically significant in this study.

### Ethical consideration

Ethical clearance was obtained from Jos University Teaching Hospital Institutional Health Research Ethical Committee prior to the commencement of the study. Written and verbal informed consents were obtained from all the respondents with confidentiality and anonymity of their responses assured and maintained.

## RESULTS

Most (78.2%) of the respondents in this study were older than 25 years of age with a mean age of 34 ± 10 years. With regards to the educational status of the commercial tricycle operators, 141 (64.1%) had completed secondary level of education and 151 (68.6%) had ever been married. Assessment of the number of hours put into operating commercial tricycle daily revealed that 179 (81.45) of the respondents had put in more than 8 hours of work per day with a mean duration of 10.7 ± 2.3 hours per day. Furthermore, 50 (22.7%) affirmed to a positive history of use of psychoactive substance by their parent or an immediate member of their families [Table 1].

Psychoactive substance was found to be currently used by 95 (43.2%) of the respondents while the prevalence of lifetime use of psychoactive substance use was 47.7%. Additionally, various psychoactive substances were expressed to have been used by the respondents with alcohol accounting for 61 (64.2%), cigarettes/tobacco product 31 (32.6%), marijuana 7 (7.4%) opioids 15(15.8%) and solution 4 (4.2%) respectively. Furthermore, the use of the substance were categorized into single and multiple use with 43 (45.3%) using a combination of two or more substance together and the age of substance use debut being below 18 years among 46 (48.4%) with a mean age of substance use debut being 24 ± 6 years [Table 1].

The use of psychoactive substance is a phenomenon that is usually influenced by factors intrinsic and extrinsic to the users. In the study, the odds of psychoactive substance use among the commercial tricycle operators with tertiary level of education was 0.06 times the odds of its use among those with no formal education. Furthermore, the odds of substance use among those with positive family history of substance use was five times the odds of those without such history. Additionally, the odds of use of psychoactive substance among those respondents whose partners or spouses engaged in the use of these substance was three times those whose partner or spouses did not use such having controlled for all other factors in the logistic regression model [Table 2].

## LIMITATIONS

In view of the sensitive nature of issues surrounding substance use, some of the respondents may have opted for socially and culturally acceptable responses which may not have been a true reflection of their actions regarding substance use. However, this was circumvented by ensuring that detailed explanation of the study was provided to the respondents with assurance of utmost confidentiality of information provided as well as ensuring that privacy was provided in the course of eliciting response without being judgemental. Furthermore, this study used a self-reported approach to assessing use of psychoactive substances which in itself is limited in its ability to validate the actual and level of use through other proxy means such the use of blood or urine samples as means of assessment thereby creating a need for further studies in this regard.

## DISCUSSION

Commuting is an essential part of life particularly in a metropolitan setting where vehicular movement is a vital part daily activity. This brings to bear the importance of ensuring that the operators of commercial tricycles are in sound state of mind devoid of any psychoactive substance influence. In this study, the prevalence of lifetime and current use of psychoactive substance was reported in more than a third of the subjects which was higher than findings of other studies conducted in Nigeria, Ghana and Spain.^[8,9,10,11]^ However, other studies found a much higher levels of psychoactive substance use among similar study population.^[12,13]^ The comparability of the findings of this study with others has brought to light that the safety of commuters and other road users could be negatively affected by the actions of commercial transport operators working under the influence of psychoactive substances. Furthermore, this it is expected that commercial vehicular operators should not be under the influence of any stimulant or psychoactive agent. However, findings of all these studies further reiterate that specific interventions need to be put in place to address this seemingly ill recognized public health problem in order to avert a more explosive disaster. It is important to elucidate on the fact that assessment of psychoactive substance use in the study was self-reported as against other studies where blood levels of such substances were assessed to corroborate and validate the self-reported information provided by the participants. Additionally, this study could not assess the contribution of psychoactive substance use to road traffic accidents implying that other studies should explore this so as to provide appropriate documentation for it.

The pattern of psychoactive substances used by the respondents in the study ranged from alcohol, tobacco, opioids, marijuana, heroin to a locally made inhalant referred to as “solution”. This is in synergy with findings of other studies conducted in Nigeria and outside Nigeria where similar pattern of psychoactive substance were used by commercial vehicular operators.^[4,5,8,10,14]^ Other studies reported slight variation in the pattern of psychoactive substances use which included antihistamines, sedative cough suppressants, amphetamines, benzodiazepines, cannabis and cocaine.^[12,15]^ This variation could be attributable to dissimilarity in the cultures of the settings where these studies were conducted as well as the ease of access to such substances and the probability that the type of psychoactive substance peddling networks is in existence. Furthermore, this has also brought to light the status of the law enforcement institutions on the control of illicit substances in these settings and the ability of illicit substance peddlers to outdo these law enforcement institutions thereby making these psychoactive substances available to the user. It is therefore imperative to ensure that more stringent mechanisms and measures are adapted to reducing the ease of access to these psychoactive substances and ensuring those identified users are provided with the treatment specific needs.Provision of appropriate and evidence-based intervention at addressing the illicit use of psychoactive substance should be hinged on identified predictors potentiating or negating its use. Therefore in this study, tertiary level of education was found to be a factor negating the use of these substances while a positive family history of illicit use of psychoactive substances and history of use in spouses or partners potentiate its use. Other studies reported factors such need to stay awake, suppression of fatigue, peer pressure, improvement in mood, younger age, use for coping, family history, enhancement of visibility while driving and for relaxation as potentiating the use of these illicit substances.^[3,5,16,17]^ This implies that in addressing the menace of illicit use of psychoactive substances among commercial tricycle operators and drivers at large, context specific interventions would be needed in addition to embracing other identified treatment needs of the subjects.

## CONCLUSION

This study has demonstrated a high level of illicit psychoactive substance use among commercial tricycle operators with higher level of education negatively influences its use and a positive family history and history of use in partners in spouses potentiating it. It is therefore imperative to structure appropriate home-grown intervention centred on these factors to addressing this problem.

## Figures and Tables

**Table 1:** Socio-demographic characteristics, prevalence and pattern of psychoactive substance use among the respondents

Characteristics	Frequency *n* = 220	Percentage
Age group		
≤25 years	48	21.8
>26 years	172	78.2
Level of education		
No formal	9	4.1
Primary	46	20.9
Secondary	141	64.1
Tertiary	24	10.9
Marital status		
Never married	69	31.4
Ever married	151	68.6
Duration of working per day (hours)		
≤8	41	18.6
>9	179	81.4
History of parent or immediate family member(s) using psychoactive substance		
Yes	50	22.7
No	170	77.3
History of partners/spouses using psychoactive substance		
Yes	84	38.2
No	136	61.8
History of colleagues/friends using psychoactive substance		
Yes	211	95.9
No	9	4.1
Life time use of psychoactive substance		
Use	105	47.7
Not use	115	52.3
Current use of psychoactive substance		
Use	95	43.2
Not use	125	56.8
Type of psychoactive substance currently used**		
Alcohol	61	64.2
Cigarette/tobacco	31	32.6
Marijuana	7	7.4
Heroin	1	1.1
Opioids (codeine or tramadol)	15	15.8
Kolanut	34	35.8
Caffeinated drinks	12	12.6
Inhalants	4	4.2
Categorization of psychoactive substance currently used (*n*=95)		
Single substance	52	54.7
Multiple substances	43	45.3
Age at current substance use debut (years); *n*= 95		
<18	46	48.4
≥18	49	51.6

** Multiple responses allowed.

**Table 2:** Multiple logistic regression of predictors of psychoactive substance use

Factors	COR (95% CI)	AOR (95% CI)	*P*-value*
Age group (years)			
>26	1.02 (0.5341–1.9396)	1.29 (0.5226–3.2030)	0.5776
<25	1	1	
Marital status			
Never married	1.14 (0.6374–2.0213)	1.62 (0.7086–3.6788)	0.2542
Ever married	1	1	
Duration of working per day (hours)			
9 and more	1.29 (0.6458–2.5828)	0.96 (0.4244–2.1477)	0.9109
<8	1	1	
Level of education			
Primary	0.44 (0.0767–2.4833)	0.40 (0.0637–2.5486)	0.3341
Secondary	0.32 (0.0591–1.6779)	0.23 (0.0391–1.3827)	0.1088
Tertiary	0.11 (0.0155–0.7130)	0.06 (0.0074–0.4806)	0.0081
No Formal	1	1	
Religion			
Islam	0.99 (0.5734–1.7324)	1.04 (0.5371–1.9977)	0.9163
Christianity	1	1	
Family history of substance use			
Yes	5.22 (2.5764–10.5680)	4.3110 (1.9662–9.4521)	0.0003
No	1	1	
History of substance use by partners/spouses			
Yes	3.89 (2.1922–6.9136)	3.30 (1.7164–6.3611)	0.0001
No	1	1	
History of substance use by colleagues/friends			
Yes	2.87 (0.5818–14.1220)	1.80 (0.3252–9.9366)	0.9163
No	1	1	

COR = Crude Odds Ratio, AOR = Adjusted Odds Ratio,

* *P*-value for AOR.
